# Editorial Comment to Successful recovery from coronavirus disease 2019 in a living kidney transplant recipient using low‐dose methylprednisolone

**DOI:** 10.1002/iju5.12238

**Published:** 2020-10-25

**Authors:** Toshio Takagi

**Affiliations:** ^1^ Department of Urology Tokyo Women's Medical University Tokyo Japan

Tanaka *et al*. described a case of coronavirus disease 2019 (COVID‐19) in a kidney transplant recipient who successfully recovered with low‐dose methylprednisolone therapy.^1^ The topic is interesting, as COVID‐19 cases have been rarely reported in kidney transplant recipients in Japan.

Cytokine storm triggered by COVID‐19 is reportedly associated with disease aggravation, and IL‐6 (one of the potential factors causing cytokine storm)‐suppressing agents such as tocilizumab are used in such patients with COVID‐19.^2^ Therefore, corticosteroids are expected to reduce disease aggravation for COVID‐19, despite controversial results shown in a randomized trial,^3^ as Tanaka *et al*. discussed in the manuscript.^1^

Addressing COVID‐19 in kidney transplant patients requires specific recognition, including a bit of different clinical symptoms from patients in the general population or the management of immunosuppressants. Akalin *et al*. described low‐grade fever as an initial symptom in these patients, more rapid clinical progression, and a very‐high early mortality (28% at 3 weeks) than in the general population. In that view point, kidney transplant patients must be closely monitored despite a lack of symptoms.^4^ With regard to immunosuppressants, antimetabolites such as mycophenolate mofetil is discontinued in most patients and the use of tacrolimus should be closely monitored with the use of antiviral drugs such as lopinavir/ritonavir due to drug interaction, which is reported by Kim *et al*.^5^

So far, an effective vaccine for COVID‐19 has not been developed. Even after practical use in the general population, it remains uncertain whether kidney transplant patients can receive these benefits due to their immunocompromised state.

## Conflict of interest

The author declares no conflict of interest.
